# Predicting and identifying correlates of inequalities in breast cancer screening uptake using national level data from India

**DOI:** 10.3389/frai.2025.1729796

**Published:** 2026-01-20

**Authors:** Aleena Tanveer, Raja Hashim Ali, Jitendra Majhi, Moumita Mukherjee

**Affiliations:** 1 Institute of International Health, Charité—Universitätsmedizin Berlin, Berlin, Germany; 2 Department of Business, University of Europe for Applied Sciences, Potsdam, Germany; 3 All India Institute of Medical Sciences, Kalyani, West Bengal, India

**Keywords:** accessibility, breast cancer screening, concentration index, concentration index decomposition, health inequality, India, machine learning

## Abstract

**Background:**

Despite national screening initiatives, coverage of breast cancer screening is low, and late-stage diagnosis remains a major contributor to mortality among Indian women. Accurate, precise, and actionable prediction of socioeconomic and structural inequities in screening uptake is critical for formulating equitable cancer control policies. This study aimed to apply machine learning to predict determinants of screening uptake, estimate inequalities in uptake and their concentration indices, and identify contributing factors to inequity using concentration index decomposition across economic, educational, and caste gradients.

**Methods:**

Cross-sectional National Family Health Survey (NFHS-5) 2019–2021 data, comprising 68,526 women aged 30–49 years, is used for the study. Levesque’s framework of healthcare access directed variable selection across approachability, acceptability, affordability, availability, and appropriateness dimensions to decide on the set of explanatory covariates. We applied three single learners—Logistic Regression (LR), Naïve Bayes (NB), and Decision Tree (DT)—and two ensemble learners—Random Forest (RF) and XGBoost (XGB)—to train on balanced weighted data. Given the risk of overfitting after the synthetic minority oversampling technique (SMOTE), predictive performance was validated using 10-fold cross-validation. Five evaluation metrics were compared to select the best learner predicting the screening uptake. Inequality was measured using conventional and algorithm-based concentration indices and decomposed using algorithm-based feature importance and feature-specific inequality scores to estimate contributions to three inequality-health gradients in screening access.

**Findings:**

In India, remarkably low (0.9%) screening uptake with clear economic, educational, and social disparities is evident. Although Random Forest and XGBoost performed with higher predictive accuracy (96%) and explainability (AUROC = 0.99), Decision Tree brought stable generalizability (mean AUROC = 0.995) after 10-fold validation. Feature importance results indicate that education, autonomy, interactions with community health workers, provincial and spatial features explain most of the variability. Proximity, transport availability, hesitancy in unaccompanied care seeking, and financial constraints were access barriers with limited contribution to the variation in screening uptake. Concentration index estimates reflect a pro-rich (0.1, *p* < 0.001), pro-educated (0.182, *p* < 0.001), and pro-marginalized social gradient (−0.011, *p* < 0.05). Tree-based decomposition predicts higher affordability, and education deepens pro-rich and pro-educated inequalities but can be an effective policy instrument to mitigate social position-based disparities if contributions can be increased. Access-related barriers intensified inequality across all gradients. Nevertheless, factors that enable access flatten the gradients.

**Conclusion:**

Machine learning models can improve decision making, enhancing accuracy and precision in inequity prediction for breast cancer screening uptake and revealing crucial gradients and access barriers shaping breast cancer screening uptake in India. ML-based predictions that offer higher explainability suggest that financial protection, spatial accessibility to health centers, access to education, autonomy, higher contact with community health workers, and community-based awareness programs targeting poor, less educated, socially disadvantaged middle-aged women are likely to smooth the economic, educational disparities in screening coverage, claiming a requirement of deeper investigation with respect to social gradients.

## Introduction

Late detection of breast cancer, contributing to higher death and disability, is a significant global public health priority (Bhattacharya et al., 2022). The need-based targeting of the missed hopes under the widespread screening program in India deserves accurate, precise evidence. India reported approximately 1.46 million new cancer cases, with a projection of 1.57 million by 2025 (Sathishkumar et al., 2022). Globally, breast cancer incidence is projected to increase by more than 40% from 2020 to 2040, and mortality is expected to increase by over 50%, disproportionately impacting low- and middle-income countries (Kim et al., 2025). India had 192,020 new breast cancer cases, which account for almost 28.2% of all female cancers. Similarly, India reported 98,337 deaths due to breast cancer in 2022 (Mortality to Incidence Ratio ≈ 0.512), meaning roughly one death for every two women diagnosed (Sathishkumar et al., 2022; Kim et al., 2025). Despite a higher burden of breast cancer among Indian women (21.8% of the total cancer-related Disability-Adjusted Life Years (DALYs) in 2020) with significant regional disparity (685.5 per 100,000 in northern India and 677.6 per 100,000 in the south), nationwide screening uptake remains extremely low (Jena et al., 2024; International Institute for Population Sciences - IIPS/India and ICF, 2022). Given the benefit of early detection at a localized stage, in reducing cancer mortality by up to 30% (WHO, 2022), only 0.9% of women aged 30–49 in India had ever undergone a clinical breast examination (International Institute for Population Sciences - IIPS/India and ICF, 2022). Tamil Nadu had the highest rate at just 5.6%, and many states reported rates well below 1% (Gopika et al., 2022).

In India, the high cancer burden is attributed to lifestyle changes with varying prevalence by socioeconomic and demographic factors, including education, caste, rural residence, and access dimensions (Smith and Mallath, 2019; Aashima and Sharma, 2024; de Siqueira Filha et al., 2022; George et al., 2020). For example, indigenous women still avoid healthcare due to historical discrimination and a lack of culturally appropriate services (George et al., 2020). Under India’s National Program for Non-Communicable Diseases (NP-NCD), breast cancer screening is delivered via Clinical Breast Examination (CBE) at Health and Wellness Centers. CBE is free of cost and recommended every 5 years for women aged 30–65. Unlike high-income countries, India does not offer population-wide mammography; thus, uptake depends heavily on the availability of trained providers, outreach, and system readiness. An observational cross-sectional study by Negi and Nambiar (2021) using NFHS-4 data on 699,686 women reported that socioeconomic-related inequalities in breast cancer screening coverage were most evident among Christians (Slope Index of Inequality, or SII: 20.6, 95% CI: 18.5–22.7), currently married (SII: 14.1, 95% CI: 13.8–14.4), employed (SII: 14.6, 95% CI: 13.9, 15.3), and rural women (SII: 10.8, 95% CI: 10.5–11.1) compared to their urban, richer, and socially advantaged counterparts measuring the Slope Index of Inequality (SII) (Negi and Nambiar, 2021). Another study assessing the prevalence of cervical cancer screening among women aged 30 to 49 (*n* = 357,353) using NFHS-5 found wealth-based inequality patterns in screening prevalence (Muthuramalingam and Muraleedharan, 2023). Rahman et al. (2025) reported that the uptake of breast cancer and cervical cancer screening tests was low, at 9 and 20 per 1,000 women, respectively and higher among the richest (Ref. Poorest; SII: 1.1 [breast cancer], 1.8 [cervical cancer]), more pronounced in rural areas (Ref. Urban; Relative Concentration Index or RCI: 22.5 [breast cancer], 21.3 [cervical cancer]), educated women (Ref. No education; relative index of inequality or RII: 4.84 [breast cancer], 2.12 [cervical cancer]). The Northeastern region showed greater socioeconomic inequality (RCI: 32.8), while the Western region showed more education-based inequality (RCI: 30.9) using relative CI (RCI). According to NFHS-5 data, uptake of breast cancer examination was higher in the Southern region, with 23.7 breast cancer examinations per 1,000 women (95% CI: 22.7–24.8), and urban women were more likely to get screened than rural women, with 12.8 breast cancer examinations per 1,000 women (95% CI: 12.1–13.4) (Rahman et al., 2025). A systematic review found that lack of awareness, long waiting times, unavailability of timely appointments, and distance to facilities limit people’s ability to approach screening. Furthermore, social embarrassment, unavailability of female healthcare staff, fear of screening or diagnosis, and cultural beliefs hindered acceptability. Other barriers included insufficient availability of services, affordability issues, and poor appropriateness, failing to match patients’ needs. Additionally, lack of spousal or family support, anxiety, and psychosocial fear further reduced uptake of screening (Srinath et al., 2023).

Despite policy efforts to improve cancer screening coverage in India, significant disparities persist in access to these services, and a detailed predictive analysis of inter-group disparities in screening access remains scarce. The Government of India has responded with programs like the National Program for Non-Communicable Diseases (NP-NCD) and the Ayushman Bharat Health and Wellness Centers (HWCs), rendering population-based screening services with a plan of 200 district-level Day Care Cancer Centers, and exempting 36 lifesaving cancer drugs from customs duties to improve affordability (Press Information Bureau, 2024). Socioeconomic inequities based on caste, economic status, and education continue to shape healthcare access, particularly for preventive services like screening (Negi and Nambiar, 2021). These disparities are neither uniform nor well understood across the country. Limited availability of evidence-based support curbs the effect size of these interventions (Chanakira et al., 2024). Therefore, robust prediction of breast cancer screening uptake, identification of crucial factors that determine the likelihood of uptake, and the underlying inequalities, when applying the most effective tool, are major public health priorities in a resource-scarce setting.

For the last couple of years, studies show that machine learning performs predictions of health service uptake and identifies important predictors or features with higher accuracy and precision in comparison to conventional modelling techniques (Baykemagn et al., 2025; Fahey et al., 2022; Ijaiya et al., 2025; Zegeye et al., 2025). Analysis of a weighted DHS, 2022 dataset of 4 sub-Saharan African nations comprising 33,952 participants, inferred that random forest (RF) performed the best with 78% accuracy and 86% explainability (area under the receiver operating characteristic curve or AUROC) in identifying the predictors of cervical cancer screening uptake (Baykemagn et al., 2025). Likewise, the study using weighted data of 299,759 respondents from DHS sub-Saharan Africa again found RF as the best algorithm (83% accuracy, 89% AUROC) in predicting homebirth and identifying its determinants (Zegeye et al., 2025). Another study applied programmed data of four Nigerian states on 41,394 patients to predict interruptions in treatment among people living with HIV on antiretroviral therapy, who found AdaBoost as the best performer (AUROC = 84%) (Ijaiya et al., 2025). Similarly, a study applied an ensemble decision tree to electronic medical record data and randomized control trial survey data from 184 patients over time. It achieved a mean accuracy of 75.2% in predicting the risk of disengagement (Fahey et al., 2022). Studies focusing on the Indian subcontinent depict the success of machine learning in the prediction of cancer screening uptake (Chandar et al., 2025; Kolasseri and Bhimavarapu, 2025). A longitudinal cancer prevention program (population-based: 14,801 participated in the screening out of 57,270 invited individuals) in India found machine learning models predicting screening uptake with >70% accuracy. The study reflects the prospective future of ML to tailor public health programs and improve screening uptake. In another study, weighted RF performed the best in predicting the determinants of cervical cancer screening uptake in India using NFHS 4 data, with 94% accuracy and 70% AUROC (Kolasseri and Bhimavarapu, 2025). Furthermore, different studies successfully applied feature importance analysis, like Shapley Additive Explanations (SHAP), permutation importance analysis to identify the determinants of service uptake or risk factors after ML prediction. These studies indicate the utility of these tools in public health decision making (Hashtarkhani et al., 2025; Ijaiya et al., 2025; Kolasseri and Bhimavarapu, 2025; Saha et al., 2023; Sultana et al., 2025). Access to mammography, educational level, affordability, health visits, and proximity to a health center are likely to explain the most variance in cancer screening uptake, as per the findings (Arage et al., 2025; Hashtarkhani et al., 2025; Kolasseri and Bhimavarapu, 2025).

Given this backdrop, current research seeks to uncover the predictors, extent, and nature of inequity in access to breast cancer screening in India, using the nationally representative National Family Health Survey (Round 5) (An excerpt of literature review matrix is presented in Table 1). Our study brings novelty in predicting and exploring the determinants and performing decomposition of CI from population-based survey data, applying ML for more accurate prediction and feature identification for both the breast cancer screening uptake and inequality in uptake. Since the data were not collected through a breast cancer–specific intervention or a randomized controlled trial, deriving insights with higher accuracy using conventional modeling techniques is challenging. Our study is the first attempt to explore this direction with respect to breast cancer screening uptake among 30–49-year-old Indian women using DHS data. This study has a triad of aims: (1) prediction of screening uptake using ML models incorporating basic sociodemographic and accessibility factors, (2) estimation of CI as a measure of inequities by different rank variables using conventional methods and ML, and (3) prediction of structural determinants contributing to inequality in uptake through ML-based CI decomposition.

**Table 1:** Literature review matrix.

Author (Year)	Location	Focus/Objective	Key findings	Limitations
Negi and Nambiar (2021)—*BMC Women’s Health*	India (NFHS data)	Distribution of socioeconomic inequalities in Breast Examination (BE) coverage	Wealth-related inequalities in BE coverage were evident, favoring wealthier women	Limited to women aged 15–49; no distinction between screening and diagnostic exams
Hailegebireal et al. (2024)—*Frontiers in Public Health*	Sub-Saharan Africa	Prevalence and predictors of Clinical Breast Examination (CBE)	Overall prevalence of CBE was low; socioeconomic and educational factors strongly influenced uptake	Self-reported responses → recall and social desirability bias
Muthuramalingam and Muraleedharan (2023)—*BMC Women’s Health*	India	Prevalence and wealth-based inequality in cervical cancer screening	Screening is significantly higher among wealthier, educated, urban women	Limited information on types of tests; inequalities underexplored in rural populations
Rahman et al. (2025)—*Journal of Epidemiology*	India	Regional variations in the uptake of breast cancer early detection	Very low overall uptake of BCE; marked inequalities between states and socioeconomic groups	Outcome measurement is subject to recall bias
Sawhney et al. (2023)—*BMC Cancer*	India	Socio-cultural barriers to seeking early detection for breast cancer	Women with higher awareness and participation in self-help groups accessed screening more	Conducted in Mumbai (urban setting) → not generalizable to rural India
Arage et al. (2025)—*BMC Medical Informatics and Decision Making*	10 Sub-Saharan African countries (DHS data)	Predict cervical cancer screening uptake using ML and identify determinants	Extra Trees & Random Forest performed best (accuracy ≈94%). Key predictors: health-facility visits, proximity, contraceptive use, residence, and media exposure.	Cross-sectional data (no causality), self-report bias, lacks psychosocial/behavioral variables, potential overfitting, and limited generalizability.
Baykemagn et al. (2025)—*JMIR Public Health and Surveillance*	Sub-Saharan Africa (DHS dataset)	Predictors of cervical cancer screening uptake using machine learning	Random Forest and Extra Trees performed best (AUC ≈ 0.86). Major predictors: age at first sex, HIV testing, STI awareness, education, wealth, and residence.	Cross-sectional design, limited variables (no attitudinal/behavioral data), self-report, no external validation.
Chandar et al. (2025)—*ASCO Journal of Clinical Oncology, Abstract e22523*	India, rural subdistrict (256 villages)	Apply ML to predict uptake and non-uptake of free population-based oral, breast, and cervical cancer screening	ML models achieved >70% accuracy and Brier Score <0.2 (except Neural Network). Nine sociodemographic/personal variables significantly correlated with screening uptake.	Limited to one rural district; no model metrics beyond accuracy; short observation window; conference abstract (no peer-reviewed publication); unknown external validity.
Saha et al. (2023)—*PLOS Global Public Health*	Southern Bangladesh (hospital-based SLE clinic)	Use ML (RF, LR) to identify predictors of vitamin D insufficiency in SLE patients	RF outperformed LR; hemoglobin, CRP, ESR, and age were the most predictive; improved model explainability.	Small sample (n = 50); cross-sectional; single-center; overfitting risk; not generalizable.
Sultana et al. (2025)—*PLOS Global Public Health*	Bangladesh (BDHS 2022)	Predict low birth weight and rank top predictors using ML (XGBoost)	XGBoost achieved ~80% accuracy (AUC ≈ 0.76). Key factors: pregnancy duration, ANC visits, C-section, delivery place, and marriage-to-first-birth interval.	Cross-sectional DHS (recall bias), limited clinical detail, missing gestational-age data, and no external validation.
Fahey et al. (2022)—*PLOS Global Public Health*	Tanzania (HIV care clinics)	Predict disengagement from HIV care using time-varying EMR data	EMR-only model accuracy ≈75%, sensitivity ≈55%; top predictors were treatment changes, weight, WHO stage.	Small cohort (n = 178); moderate sensitivity; lacks psychosocial/contextual predictors; no external validation.
Ijaiya et al. (2025)—*PLOS Global Public Health*	Nigeria (four states; HIV program EMR)	Predict interruption in HIV treatment using ML models	AdaBoost achieved sensitivity ≈69%, specificity ≈82%, AUC ≈ 0.84; prior behavior and geography strongest predictors.	Dataset-specific; EMR quality concerns; limited contextual variables; no prospective validation.

## Methods

NFHS is cross-sectional, nationally representative, and conducted every 5 years under the Demographic and Health Survey (DHS) program, which collects information on demographic, reproductive, maternal, and child health, nutrition, household characteristics, and gender-based violence using structured questionnaires. The NFHS-5 surveyed 724,115 women aged 15–49 years, yet in line with ICMR’s cancer screening policy, this evaluation is limited to 68,526 participants, including women aged 30–49 years, mirroring the approach in similar population studies (Rahman et al., 2025). According to the Indian Government’s operational framework, CBE is recommended for all women between 30 and 65 years of age at five-year intervals (Ministry of Health & Family Welfare Government of India, 2023). The reason for excluding women under 30 lies in the fact that the incidence of breast cancer before 30 years of age is low, and screening of younger women with clinical breast exam or imaging is not validated or cost-effective in the context of the Indian population (Khanna et al., 2024). This study follows the CLAIM reporting checklist for AI/machine learning in health research. A completed checklist is provided in Supplementary Table 1.

### The theoretical framework and variable description

**Figure 1:**
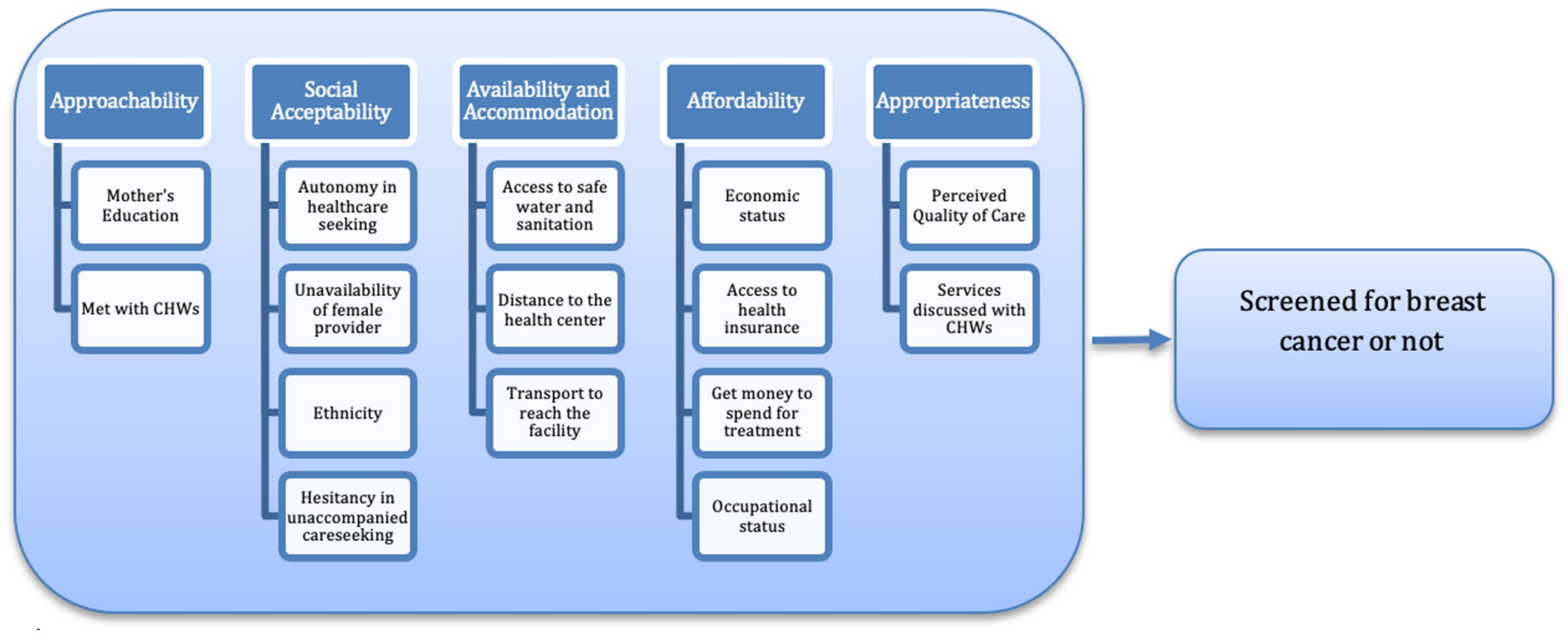
The theoretical framework of healthcare access and its correlates, following Levesque et al. (2013).

From NFHS data, we used select variables about the theoretical framework, the Levesque model (Figure 1) (Levesque et al., 2013). The ability to perceive determines *approachability* and is measured by health literacy, health beliefs, trust, and expectations (Davy et al., 2016). We estimated using proxy indicators, mothers’ education level, and whether respondents met any community health workers, as health literacy and beliefs are positively associated with one’s level of education (Hawley et al., 2008; Rostamzadeh et al., 2022) and more counselling by health workers. The ability to seek healthcare is the determining factor for *social acceptability*, measured in personal and social values, culture, gender, and autonomy (Davy et al., 2016). The proxy indicators selected are the health-seeking autonomy index (measured by obtaining permission to seek healthcare for oneself and obtaining permission from husband to give birth at a healthcare facility), unavailability of a female health provider, hesitancy in unaccompanied care, and ethnicity. The *availability* and *accommodation* in healthcare-seeking behavior are determined by the *ability to reach* healthcare centers and are measured in the living environment, transport, mobility, and social support (Levesque et al., 2013; Syed et al., 2013). The variables used are as follows: access to safe water and sanitation, distance to the health center, and transport availability to reach the health facility. *Affordability* is measured as the ability to pay and it depends on the care seeker’s income/assets and access to social capital, such as health insurance (Peters et al., 2008), measured using the indicators of whether the participant has insurance coverage, economic status, gets money to pay for healthcare (willingness to pay), and the respondent’s occupational status. Finally, the ability to engage in healthcare utilization is assessed as *appropriateness*, indicating information, adherence, caregiver support, and the care seeker’s perception about the quality of care provided. The proxy indicator for information exposure and adherence is operationalized using interactions with Community Health Workers (CHWs), including whether services were discussed and the frequency of meetings. The perceived quality of care (PQC) is used to measure care seekers’ perception of the quality of services rendered in the health centers. The PQC index is generated using variables such as facility timings, presence of healthcare personnel, long/short waiting times, and the quality of service rendered. We ran Cronbach’s *α* test to assess how well the variables considered for the constructs represent them. All variables generated and used are listed in Table 2.

**Table 2:** Variable description.

Dependent variable	Type	Categories
Ever undergone a breast examination for breast cancer?	Binary	Yes
		No

Features	Type	Description
Sociodemographic factors		
Socioeconomic Status	Ordinal	Poorest
		Poorer
		Middle
		Richer
		Richest
Ethnicity	Nominal	Caste
		Tribe
		No cast/tribe
		Do not know
Education level	Ordinal	No Education
		Primary
		Secondary
		Higher
Age groups	Categorical	30–34
		35–39
		40–44
		45–49
Theoretical framework		
Approachability factors		
Mother’s education level	Ordinal	No Education
		Primary
		Secondary
		Higher
Met with CHW	Nominal	Met none
		Met any 1 type
		Met any 2 types
		Met any 3 types
Type of CHW met	Nominal	Met none
		Met ANM
		Met AWW
		Met any ASHA
		Met MPW
		Met Other
Social acceptability factors		
Unavailability of a female provider	Binary	No problem
		Problem
Autonomy in healthcare seeking	Binary	Allowed to seek care
		Not allowed to seek care
Ethnicity	Nominal	Caste
		Tribe
		No cast/tribe
		Do not know
Hesitancy in unaccompanied care seeking	Binary	No problem
		Problem
Availability and accommodation factors		
Access to safe water	Categorical	Piped water
		Tube well
		Well
		Spring water
		Rain water
		Bottled water
		Others
Access to a sanitation facility	Binary	No flush toilet
		Flush toilet
Distance to the health center	Binary	No problem
		Problem
Transport to reach the facility	Binary	No problem
		Problem
Affordability factors		
Economic status	Ordinal	Poorest
		Poorer
		Middle
		Richer
		Richest
Access to health insurance	Binary	No
		Yes
Get money to spend on treatment	Binary	No problem
		Problem
Occupational status	Binary	Unemployed
		Employed
Appropriateness factors		
Services discussed with CHWs	Nominal	No services discussed
		CHW discussed disease prevention, treatment, and health education
Perceived Quality of Care Index (PQC)	Binary	Low PQC
		High PQC

We pre-processed the data before analysis, checking for null values and distributional patterns to assess the need to impute/truncate values or transform any variable. Secondly, given the data imbalance, the SMOTE oversampling technique was applied to the weighted data in Python version 3 to balance the cardinality of the outcome variable. The whole analysis was conducted on weighted resampled data.

**Figure 2:**
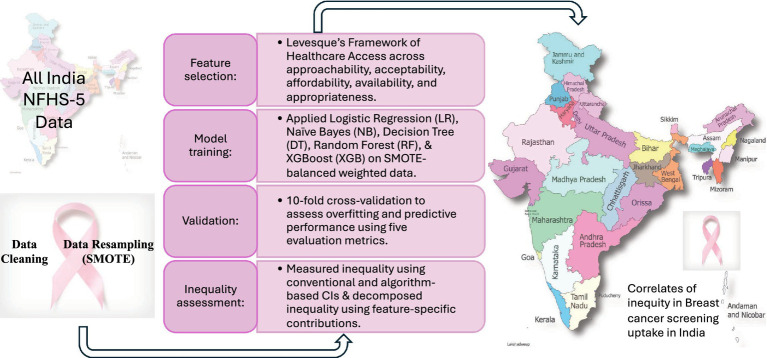
Workflow diagram.

The chi-squared (χ^2^) test of independence explored the associations between the factors under the theoretical framework and the weighted prevalence of cancer screening from the observed data. Under predictive analysis, 5 ML models—LR (base), NB, XGB, DT, and RF—are run with an 80:20 train-test split with 10-fold cross-validation to check for overfitting risks on weighted resampled data. ML models are run in 2 phases to predict the breast cancer screening uptake. The base model includes only economic, educational, and social status, which are later considered as rank factors in inequity estimation. The final model includes accessibility indicators to explore if any mediation effect exists. Therefore, the base model included only socioeconomic factors. The final model added accessibility variables to test mediation effects. We applied permutation importance and Shapley Additive Explanations (SHAP) to identify the features that contributed most to the variation in breast cancer screening uptake. Then we computed inequalities in screening uptake from observed data using standard concentration indices (CI) and from the predicted probabilities obtained by applying ML with rank factors, economic status, education level, and ethnicity, following O'Donnell et al. (2008). To further predict the sources of inequality, we performed a SHAP decomposition of CIs after using a single learner tree-based algorithm. The decomposition estimates the likely contributions of the socioeconomic, demographic, and accessibility characteristics to economic, educational, or ethnic group-specific inequalities. We estimated the predictors’ contribution to CI as the ‘feature importance’ times ‘feature-specific inequalities’ for each feature at each rank variable (economic status, education, ethnicity). The workflow is explained in Figure 2.

### Ethical considerations

We used secondary available Indian Demographic and Health Survey data, namely, National Family Health Survey round 5 (2019–21), which is accessible in the public domain. Data is available at https://dhsprogram.com/data/available-datasets.cfm. As we have not collected any primary data for the current study, we do not require any ethical approval. Therefore, institutional review committee approval is not applicable here.

## Results

### Respondent’s profile

Bivariate associations between respondents’ characteristics and screening uptake are presented in Table 3. Across India, the uptake of breast cancer screening was distinctly higher among women from wealthier quintiles and the educated subgroup. Screening rates vary from 0.31% among the poorest to 0.75% among the richest (χ^2^ = 38.9, *p* < 0.001). Similarly, screening increased sharply with education, from 0.24% in the women without education to 1.2% among women with higher educational attainment (χ^2^ = 149.2, *p* < 0.001). Caste-based disparities were also evident from the observed data, with women from advantaged groups reporting higher screening uptake compared to marginalized women (χ^2^ = 9.3, *p* = 0.026). Among health service access-related determinants, significantly associated major barriers observed are meeting with community health workers (CHWs) (higher among those who met any 1 type of CHW; χ^2^ = 59.4, *p* < 0.001), preference of female care providers (absence is a significant concern; χ^2^ = 9.1, *p* = 0.003), proximity and transport to health facilities (distance with limited transport availability affects; *p* < 0.001), financial restraints (χ^2^ = 20.1, *p* < 0.001), and perceived quality of care (χ^2^ = 6.9, *p* = 0.009). Screening access varies significantly by access to a piped water supply, to unsafe water sources, or access to a flush toilet (*p* < 0.001). Notably, women who reported no approachability, availability, accommodation, affordability, or appropriateness issues were more likely to screen. These findings call attention to structural, social, economic, and information-related inequities shaping the use of a vital preventive service.

**Table 3:** Participants’ profiles and bivariate associations between participants’ characteristics and breast cancer screening uptake.

Sociodemographic factors		National average (India)		
Variable	Variable categories	Column frequencies (%)	Screened for breast cancer, *n* (%)	Chi^2^ (*p* value)
Wealth quantile	Poorest	20,006 (29.19)	61 (0.31)	38.9 (<0.001)
	Poorer	14,118 (20.60)	54 (0.39)	
	Middle	11,461 (16.73)	59 (0.52)	
	Richer	11,151 (16.27)	73 (0.67)	
	Richest	11,790 (17.21)	85 (0.75)	
Education level	No education	21,512 (31.39)	50 (0.24)	149.2 (<0.001)
	Primary education	9,277 (13.54)	22 (0.24)	
	Secondary education	26,692 (38.95)	132 (0.50)	
	Higher education	11,045 (16.12)	128 (1.20)	
Ethnicity	Caste	51,274 (74.82)	263 (0.52)	9.3 (0.0260)
	Tribe	13,533 (19.75)	51 (0.38)	
	No caste	3,406 (4.97)	14 (0.42)	
	Do not know	313 (0.46)	4 (1.34)	

Theoretical framework variables		National average (India)		
Variable	Variable categories	Column frequencies (%)	Screened for breast cancer, n (%)	Chi^2^ (*p* value)
Met with CHW	Met none	31,530 (46.01)	143 (0.46)	59.4 (<0.001)
	Met any 1 type	12,295 (17.94)	87 (0.72)	
	Met any 2 types	14,843 (21.66)	70 (0.48)	
	Met any 3 types	9,224 (13.46)	31 (0.34)	
Type of CHW met	Met none	31,530 (46.01)	143 (0.46)	1.9 (0.8642)
	Met ANM	24,293 (35.45)	127 (0.53)	
	Met AWW	7,120 (10.39)	36 (0.51)	
	Met any ASHA	5,462 (7.97)	26 (0.48)	
Unavailability of a female provider	No problem	25,376 (37.03)	149 (0.60)	9.1 (0.0030)
	Problem	43,150 (62.97)	183 (0.43)	
Autonomy in healthcare seeking	Allowed to seek care	43,258 (63.13)	212 (0.50)	0.1 (0.8220)
	Not allowed to seek care	25,268 (36.87)	120 (0.48)	
Hesitancy in unaccompanied care seeking	No problem	32,310 (47.15)	171 (0.54)	2.71 (0.1000)
	Problem	36,216 (52.85)	161 (0.45)	
Access to safe water	Piped water	30,600 (44.65)	170 (0.57)	70.3 (<0.001)
	Tube well	23,979 (34.99)	72 (0.31)	
	Well	5,152 (7.52)	34 (0.67)	
	Spring water	3,289 (4.80)	3 (0.09)	
	Rainwater	1,383 (2.02)	13 (0.95)	
	Bottled water	1,295 (1.89)	16 (1.27)	
	Others	1916 (2.80)	12 (0.64)	
Access to a sanitation facility	No flush toilet	25,380 (37.04)	88 (0.35)	15.9 (<0.001)
	Flush toilet	43,145 (62.96)	244 (0.58)	
Distance to the health center	No problem	26,321 (38.41)	162 (0.63)	15.4 (<0.001)
	Problem	42,205 (61.59)	170 (0.41)	
Transport to reach the facility	No problem	27,325 (39.88)	165 (0.62)	13.5 (<0.001)
	Problem	41,201 (60.12)	167 (0.41)	
Access to health insurance	No	48,345 (70.55)	244 (0.51)	1.4 (<0.001)
	Yes	20,181 (29.45)	88 (0.44)	
Get money to spend on treatment	No problem	30,674 (44.76)	189 (0.63)	20.1 (<0.001)
	Problem	37,852 (55.24)	143 (0.38)	
Occupational status	Unemployed	7,185 (68.66)	34 (0.48)	0.0 (0.8755)
	Employed	3,279 (31.34)	17 (0.53)	
Services discussed with CHWs	No services discussed	59,535 (86.88)	266 (0.46)	12.4 (<0.001)
	CHW discussed disease prevention, treatment, and health education	8,991 (13.12)	66 (0.74)	
Perceived Quality of Care Index (PQC)	Low PQC	18,900 (27.58)	113 (0.61)	6.9 (0.0086)
	High PQC	49,626 (72.42)	219 (0.45)	

**Figure 3:**
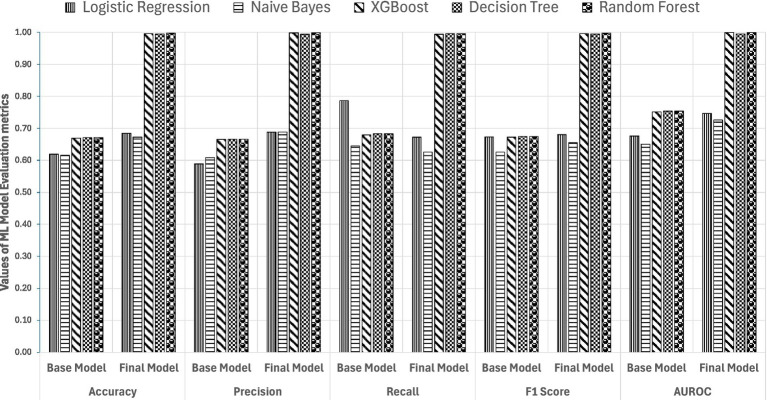
Performance analysis of 5 ML models predicting breast cancer screening uptake.

**Figure 4:**
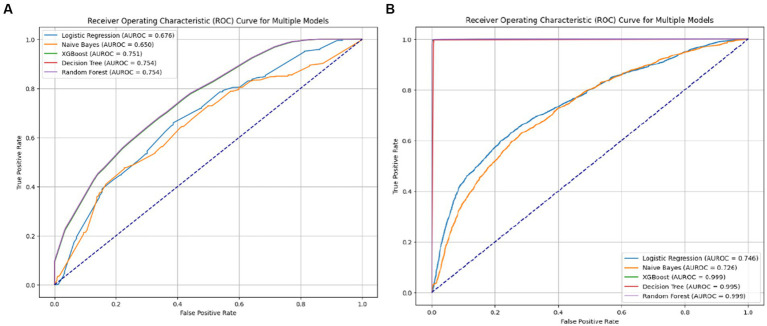
AUROC of base **(A)** and final **(B)** models.

Among the 5 ML algorithms tested, RF, XGB, and DT performed better than LR and NB (Figure 3). Predictive accuracy and model explainability (AUROC) improved sharply from the base model to the final model, indicating a prominent mediation effect of accessibility factors (Figures 4A,B). Although sensitivity is compromised in the final models of LR and NB, the F1 score improves. In relation to the discrimination ability of the models denoted by AUROC, XGB (0.999) and RF (0.999), which reflect a greater risk of overfitting that might arise after SMOTE. Compared with ensemble learners, the DT algorithm indicates robust discrimination between screened and unscreened women. Furthermore, to eliminate the risk of overfitting bias, a split analysis is conducted to identify the most stable algorithm among XGB, DT, and RF and is presented in the next section.

Model stability analysis indicates that the DT (AUROC between 0.995 and 0.996) classifier shows greater stability and generalizability compared to RF and XGB, with low SD and moderate mean value across splits for all the metrics (Table 4). In each split, the DT classifier is comparatively stable (accuracy = 0.995, AUROC = 0.995), whereas XGB shows moderate, and RF depicts a greater risk of overfitting (Table 5). A consistently low standard deviation of metrics denotes internal validity and robustness, confirming model performance was not sample-dependent. Therefore, a single tree-based learner was found to be suitable for predicting inequality of highly skewed health service uptakes.

**Table 4:** Model stability analysis—mean and standard deviation of metrics across splits.

Model	Accuracy		Precision		Recall		F1 score		AUROC	
	Mean	SD	Mean	SD	Mean	SD	Mean	SD	Mean	SD
Decision tree	0.995	0.000	0.994	0.001	0.996	0.000	0.995	0.000	0.995	0.000
Random forest	0.997	0.000	0.998	0.000	0.996	0.000	0.997	0.000	1.000	0.000
XGBoost	0.996	0.000	0.998	0.001	0.995	0.001	0.996	0.000	0.999	0.000

**Table 5:** RF, DT, and XGB classifier performance in 10 data splits.

**Split**	**Model**	**Accuracy**	**Precision**	**Recall**	**F1 Score**	**AUROC**
1	RandomForestClassifier	0.997	0.999	0.996	0.997	0.999
	DecisionTreeClassifier	0.995	0.994	0.996	0.995	0.995
	XGBClassifier	0.996	0.999	0.994	0.996	0.999
2	RandomForestClassifier	0.997	0.998	0.996	0.997	1.000
	DecisionTreeClassifier	0.994	0.993	0.995	0.994	0.995
	XGBClassifier	0.996	0.998	0.993	0.995	0.999
3	RandomForestClassifier	0.997	0.998	0.996	0.997	0.999
	DecisionTreeClassifier	0.995	0.994	0.996	0.995	0.995
	XGBClassifier	0.997	0.997	0.996	0.997	1.000
4	RandomForestClassifier	0.997	0.998	0.997	0.997	1.000
	DecisionTreeClassifier	0.995	0.994	0.996	0.995	0.996
	XGBClassifier	0.996	0.997	0.995	0.996	0.999
5	RandomForestClassifier	0.997	0.999	0.996	0.997	0.999
	DecisionTreeClassifier	0.995	0.994	0.995	0.995	0.995
	XGBClassifier	0.996	0.998	0.995	0.996	0.999
6	RandomForestClassifier	0.998	0.999	0.997	0.998	1.000
	DecisionTreeClassifier	0.996	0.995	0.996	0.996	0.996
	XGBClassifier	0.996	0.998	0.994	0.996	1.000
7	RandomForestClassifier	0.997	0.998	0.997	0.997	1.000
	DecisionTreeClassifier	0.994	0.993	0.996	0.994	0.995
	XGBClassifier	0.997	0.997	0.996	0.997	0.999
8	RandomForestClassifier	0.997	0.998	0.997	0.997	1.000
	DecisionTreeClassifier	0.995	0.993	0.997	0.995	0.996
	XGBClassifier	0.996	0.997	0.996	0.996	1.000
9	RandomForestClassifier	0.997	0.998	0.996	0.997	1.000
	DecisionTreeClassifier	0.995	0.994	0.996	0.995	0.995
	XGBClassifier	0.996	0.998	0.993	0.996	0.999
10	RandomForestClassifier	0.997	0.998	0.997	0.997	0.999
	DecisionTreeClassifier	0.994	0.992	0.996	0.994	0.995
	XGBClassifier	0.996	0.998	0.995	0.996	0.999

### Enablers and barriers of breast cancer screening uptake

**Figure 5:**
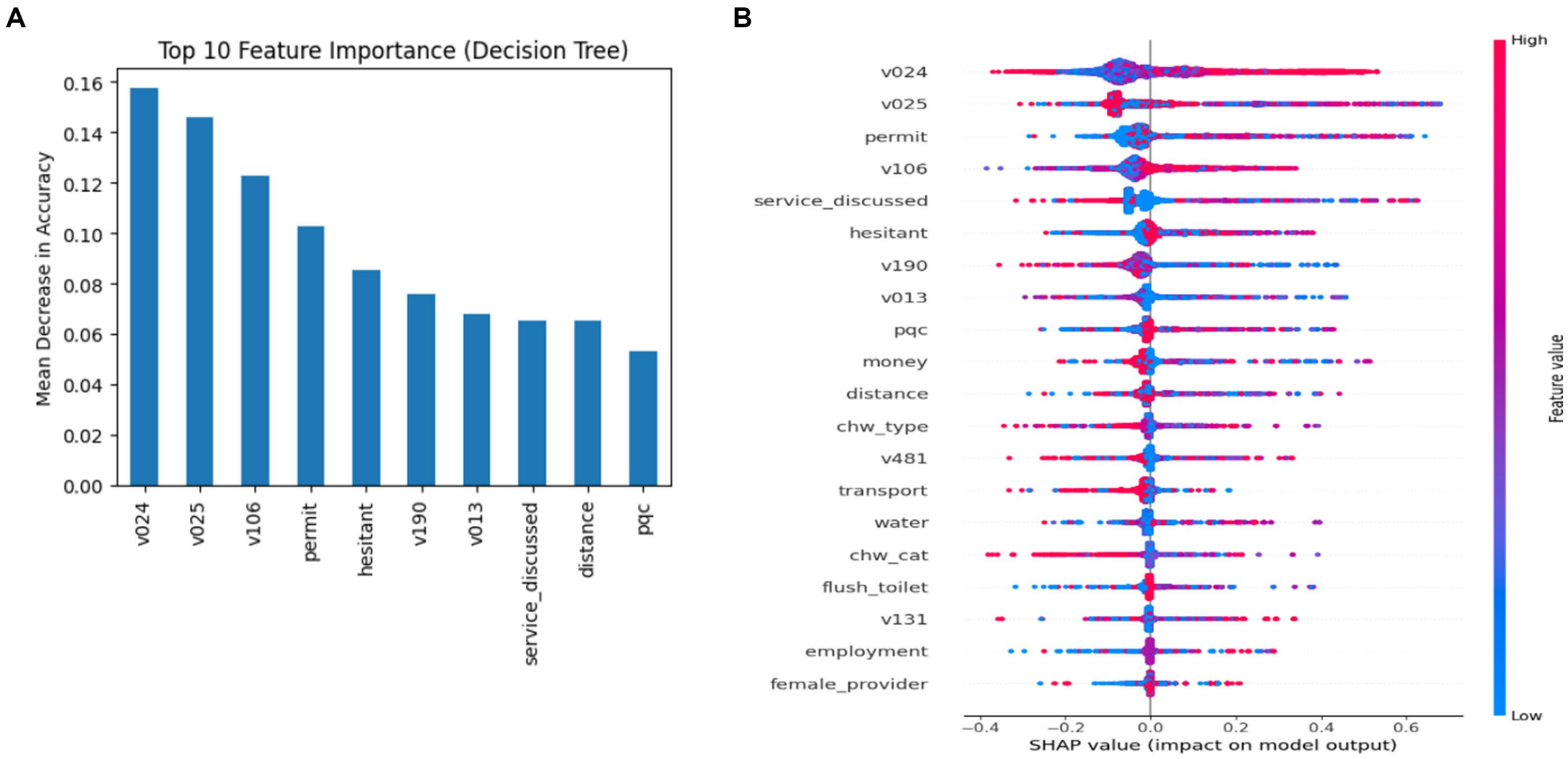
**(A)** Permutation importance and **(B)** SHAP (DT classifier).

Permutation importance (Figure 5A) and SHAP analysis (Figure 5B) reflect that socioeconomic status and the degree of health service access are important predictors of screening uptake. The strongest contributors to variability in breast cancer screening uptake among 30–49-year-old women in India, as per mean absolute SHAP values (Table 6), are state of residence (0.110) and rural or urban location of residence (0.100), degree of autonomy to seek healthcare (0.064), level of education (0.056), whether they had the opportunity to meet CHWs and received information on available services in health centers (0.053), hesitancy in unaccompanied care seeking (0.039), socioeconomic status (0.034), age of the participant (0.031), perceived quality of care provided in the health service delivery center (0.026), getting money to spend for treatment (0.026), and proximity to care (0.022). Therefore, these factors are strong structural and behavioral predictors explaining the variance. The permutation feature importance reflects a similar feature ranking with a few rank alternations at a marginal level difference. The importance score is relatively higher under permutation importance than under SHAP (Table 6). For example, the top-ranking determinants by mean SHAP and permutation importance were state (0.110, 0.157) and location of residence (0.100, 0.146), depicting high provincial and spatial variability in screening uptake. In parallel, structural determinants—proximity, appropriateness, access to information through CHWs, health-seeking autonomy, and educational attainment—also had meaningful predictive weights, indicating behavioral and informational determinants of inequality.

**Table 6:** Feature ranking by importance score under permutation importance and SHAP.

Feature	Mean absolute SHAP value	Permutation importance score
State	0.110	0.157
Type of place of residence	0.100	0.146
Autonomy in healthcare seeking	0.064	0.103
Education level	0.056	0.123
Services discussed with CHWs	0.053	0.065
Hesitancy in unaccompanied care seeking	0.039	0.085
Economic status	0.034	0.076
Age of the respondent	0.031	0.068
Perceived quality of care index	0.026	0.053
Getting money to spend on treatment	0.026	0.028
Distance to the health center	0.022	0.065

Table 7 presents concentration indices from observed values following the conventional method and decision tree-based predicted probabilities. Across three rank factors, socioeconomic and educational gradients reflect strong pro-rich (0.1675***; 95% CI [0.1302, 0.2477]) and pro-educated (0.2608***; 95% CI [0.2631, 0.3814]) inequality in screening uptake. Additionally, screening uptake is concentrated among socially disadvantaged women (−0.1455*; 90% CI [−0.1973, −0.0937]). Decision tree-based CI estimates yielded similar patterns but with attenuated magnitudes, capturing nonlinearity in economic/education/social-health gradients. Next, the decomposition analysis reveals the nature of barriers faced by poor, less educated, and potentially enabling factors that would facilitate screening among them, as well as socially disadvantaged women in breast cancer screening uptake.

**Table 7:** Concentration indices by three rank factors.

Rank factor/variable	Concentration Index using conventional regression	Concentration index using decision tree
Economic status	0.1675*** SE [0.0308] 95% CI [0.1302, 0.2477]	0.1079*** SE [0.0036] 95% CI [0.1009, 0.1149]
Education level	0.2608*** SE [0.0304] 95% CI [0.2631, 0.3814]	0.1823*** SE [0.0036] 95% CI [0.1745, 0.1882]
Ethnicity	−0.1455* SE [0.0315] 90% CI [−0.1973, −0.0937]	−0.0111** SE [0.0035] 95% CI [−0.0180, −0.0047]

* *p* < 0.1; ** *p* < 0.05; *** *p* < 0.01.

Table 8 represents the decomposition of inequality in breast cancer screening uptake using the DT algorithm by three rank factors—socioeconomic status, educational attainment, and ethnicity. Each table subsection lists the specific contribution of each rank variable’s individual factor to the overall inequity (CI) estimated as the product of feature importance and the concentration index of the respective feature. The table reports the standard errors (SE), 95% confidence intervals, and *p-*values of all estimates. Socioeconomic conditions, access to a good living environment, living in a rural area, and education accentuate pro-rich inequality [economic status (0.017***), water (0.143***), sanitation (0.221***), education (0.016***), urban residence (−0.001***)] across the economic-health gradient. Pro-educated inequality is aggravated by similar factors—economic status (0.010***), education (0.026***), access to water (0.002***), sanitation (0.001***), and urban residence (−0.006***). Conversely, these factors, along with state of residence, attenuate social position-related screening inequality [economic status (−0.001***), education (−0.002***)], favoring the general caste. It reflects the narrowing of caste-based disparities and an increase in uptake among general caste women. On the other hand, CI decomposition contribution values for accessibility barriers indicate limited proximity to health facilities and unavailability of transport, lack of autonomy, hesitancy in unaccompanied care seeking, getting money for treatment, and rural location of residency, which exacerbate disproportionately higher burdens on poor and less educated women, increasing lower screening participation among them. Likewise, enhanced affordability (easier access to money for treatment), autonomy, higher contact with CHWs, proximity to healthcare facilities, and access to a better living environment help marginalized women’s access further. On the other hand, women from the general caste with a rich, educated background, living in an urban area, have access to safe water and sanitation, which helps to mitigate social position-based gradients in breast cancer screening. Accessibility barriers hinder the participation of poor, less educated, marginalized women and indicate that successful targeting can increase uptake in breast cancer screening. It implies a strong mediation effect in economics and education and a moderate mediation effect in the social gradient of screening coverage. The advantage of applying ML to CI decomposition is that it helps capture nonlinearity—e.g., autonomy in healthcare seeking and its role in the economic gradient of screening uptake. It emerged as an important independent predictor (SHAP = 0.089) of economic inequity in breast cancer screening uptake. SHAP’s local individual-level contribution is aggregated by socioeconomic rank to compute the CI of autonomy’s contribution. It shows that disempowerment is pro-poor and significantly higher among poorer women compared to richer women. Therefore, if autonomy is targeted and directed towards decision making for breast cancer screening among poorer women, it can flatten the economic gradient faster.

**Table 8:** Decomposition of CIs by three rank factors.

Rank variable	Feature	Feature importance	CI feature	SE	Decomposition value
Economic status	Economic status	0.055	0.3029*** [0.299, 0.307]	0.0021	0.017*** [0.017, 0.017]
	Education level	0.060	0.2583*** [0.252, 0.264]	0.003	0.016*** [0.015, 0.016]
	Ethnicity	0.021	0.000*** [0.000, 0.000]	0.000	0.000*** [0.000, 0.000]
	Age groups	0.034	−0.009*** [−0.010, −0.008]	0.001	0.000*** [0.000, 0.000]
	State	0.275	0.003 [−0.001, 0.008]	0.002	0.001 [0.000, 0.002]
	Type of place of residence	0.138	−0.067*** [−0.069, −0.065]	0.001	−0.001*** [−0.010, −0.009]
	Met with CHW	0.026	−0.042*** [−0.050, −0.033]	0.004	−0.001*** [−0.001, −0.001]
	Type of CHW met	0.021	−0.025*** [−0.034, −0.016]	0.004	−0.001*** [−0.001, 0.000]
	Unavailability of a female provider	0.004	−0.102*** [−0.108, −0.096]	0.0029	0.000*** [0.000, 0.000]
	Autonomy in healthcare seeking	0.089	−0.111*** [−0.121, −0.101]	0.005	−0.010*** [−0.011, −0.009]
	Hesitancy in unaccompanied care seeking	0.018	−0.136*** [−0.143, −0.129]	0.0036	−0.002*** [−0.003, −0.002]
	Access to safe water	0.021	0.143*** [0.119, 0.168]	0.0123	0.003*** [0.003, 0.004]
	Access to a sanitation facility	0.010	0.221*** [0.216, 0.227]	0.003	0.0022*** [0.002, 0.002]
	Distance to the health center	0.031	−0.147*** [−0.153, −0.141]	0.003	−0.005*** [−0.005, −0.004]
	Transport to reach the facility	0.010	−0.159*** [−0.166, −0.153]	0.003	−0.002*** [−0.002, −0.002]
	Access to health insurance	0.015	−0.042*** [−0.054, −0.030]	0.005	−0.001*** [−0.001, 0.000]
	Getting money to spend on treatment	0.028	−0.172*** [−0.179, −0.165]	0.0035	−0.005*** [−0.005, −0.005]
	Occupational status	0.018	−0.017*** [−0.021, −0.012]	0.0022	−0.000*** [0.000, 0.000]
	Services discussed with CHWs	0.088	0.034*** [0.015, 0.054]	0.0099	0.003*** [0.001, 0.005]
	Perceived quality of care index	0.038	−0.088*** [−0.093, −0.084]	0.0024	−0.003*** [−0.004, −0.003]
Education level	Economic status	0.055	0.185*** [0.181, 0.189]	0.0021	0.010*** [0.010, 0.010]
	Education level	0.060	0.429*** [0.423, 0.435]	0.003	0.026*** [0.026, 0.026]
	Ethnicity	0.021	0.000*** [0.000, 0.000]	0.000	0.000*** [0.000, 0.000]
	Age groups	0.034	−0.015*** [−0.016, −0.014]	0.0006	−0.001*** [−0.001, −0.001]
	State	0.275	0.041*** [0.037, 0.046]	0.0022	0.011*** [0.010, 0.013]
	Type of place of residence	0.138	−0.042*** [−0.044, −0.040]	0.001	−0.006*** [−0.006, −0.006]
	Met with CHW	0.026	−0.011*** [−0.019, −0.003]	0.004	−0.0003*** [−0.0010, 0.0000]
	Type of CHW met	0.021	0.003 [−0.005, 0.012]	0.004	0.000 [0.000, 0.000]
	Unavailability of a female provider	0.004	−0.088*** [−0.094, −0.082]	0.003	0.000*** [0.000, 0.000]
	Autonomy in healthcare seeking	0.089	−0.118*** [−0.128, −0.109]	0.005	−0.012*** [−0.012, −0.010]
	Hesitancy in unaccompanied care seeking	0.018	−0.112*** [−0.119, −0.105]	0.004	−0.002*** [−0.002, −0.002]
	Access to safe water	0.021	0.096*** [0.072, 0.120]	0.0123	0.002*** [0.002, 0.003]
	Access to a sanitation facility	0.010	0.130*** [0.124, 0.136]	0.003	0.001*** [0.001, 0.001]
	Distance to the health center	0.031	−0.112*** [−0.118, −0.106]	0.003	−0.003*** [−0.004, −0.003]
	Transport to reach the facility	0.010	−0.122*** [−0.129, −0.116]	0.003	−0.001*** [−0.001, −0.001]
	Access to health insurance	0.015	0.006 [−0.006, 0.018]	0.006	0.000 [0.000, 0.000]
	Getting money to spend on treatment	0.028	−0.134*** [−0.141, −0.127]	0.004	−0.004*** [−0.004, −0.004]
	Occupational status	0.018	−0.009*** [−0.013, −0.004]	0.0022	0.000*** [0.000, 0.000]
	Services discussed with CHWs	0.088	0.057*** [0.038, 0.077]	0.0099	0.005*** [0.003, 0.007]
	Perceived quality of care index	0.038	−0.068*** [−0.073, −0.064]	0.0024	−0.0026*** [−0.003, −0.002]
Ethnicity	Economic status	0.055	−0.026*** [−0.030, −0.022]	0.0021	−0.001*** [−0.002, −0.001]
	Education level	0.060	−0.027*** [−0.033, −0.021]	0.003	−0.002*** [−0.002, −0.001]
	Ethnicity	0.021	0.000*** [0.000, 0.000]	0.000	0.000*** [0.000, 0.000]
	Age groups	0.034	0.007*** [0.006, 0.008]	0.001	0.000*** [0.000, 0.000]
	State	0.275	−0.100*** [−0.105, −0.096]	0.002	−0.028*** [−0.029, −0.026]
	Type of place of residence	0.138	0.011*** [0.009, 0.013]	0.001	0.002*** [0.001, 0.002]
	Met with CHW	0.026	−0.037*** [−0.045, −0.029]	0.0042	−0.001*** [−0.001, −0.001]
	Type of CHW met	0.021	−0.035*** [−0.044, −0.026]	0.0044	−0.001*** [−0.001, −0.001]
	Unavailability of a female provider	0.004	0.003*** [−0.003, 0.009]	0.003	0.000 [0.000, 0.000]
	Autonomy in healthcare seeking	0.089	−0.012** [−0.022, −0.002]	0.005	−0.001** [−0.002, 0.000]
	Hesitancy in unaccompanied care seeking	0.018	0.007* [0.000, 0.014]	0.004	0.000* [0.000, 0.000]
	Access to safe water	0.021	−0.080*** [−0.104, −0.056]	0.0123	−0.002*** [−0.002, −0.001]
	Access to a sanitation facility	0.010	0.021*** [0.015, 0.026]	0.003	0.000*** [0.000, 0.000]
	Distance to the health center	0.031	0.021*** [0.015, 0.027]	0.003	0.001*** [0.001, 0.001]
	Transport to reach the facility	0.010	0.020*** [0.014, 0.027]	0.003	0.000*** [0.000, 0.000]
	Access to health insurance	0.015	0.001 [−0.011, 0.013]	0.006	0.000 [0.000, 0.000]
	Getting money to spend on treatment	0.028	0.017*** [0.010, 0.024]	0.004	0.001*** [0.000, 0.001]
	Occupational status	0.018	0.006*** [0.002, 0.010]	0.002	0.000*** [0.000, 0.000]
	Services discussed with CHWs	0.088	−0.040*** [−0.059, −0.020]	0.010	−0.004*** [−0.005, −0.002]
	Perceived quality of care index	0.038	0.011*** [0.006, 0.016]	0.002	0.000*** [0.000, 0.000]

* *p* < 0.1; ** *p* < 0.05; *** *p* < 0.01.

## Discussion

Our primary objective was to achieve higher accuracy and explainability in predicting breast cancer screening uptake and capturing nonlinearity in inequality decomposition, not causal inference. In our analysis, we explored predictors of breast cancer screening uptake and inequality in uptake across India among middle-aged women aged 30–49 years using NFHS-5 data, leveraging advanced machine learning approaches such as SHAP decomposition to inform health policy aimed at increasing breast cancer screening uptake in India.

Breast cancer screening uptake markedly varies by economic status, educational attainment, and social position. Wealth and higher education remained positively associated with breast cancer screening. Screening rates of 0.75% were observed among wealthier women, compared with 0.31% in poorer ones. Screening rates of 1.2% in highly educated women, in contrast to 0.24% in those with no education. This affirms their reliability as predictors rather than chance associations consistent with national findings by Negi and Nambiar (2021) and Rahman et al. (2025). Like in Nepal, the uptake of breast cancer screening was found to be associated with occupational status, ethnicity, education, and prior information on breast cancer screening. In their study, lower odds were observed among agricultural laborers (Ref. non-working; aOR = 0.59, [95% CI: 0.42, 0.82]), higher odds among Dalit women (Ref. Brahmin/Chhetri; aOR = 2.08, [95%CI: 1.37, 3.16]), and educated women (Ref. No education; Basic: aOR = 1.49, [95%CI: 1.04, 2.13], Secondary: aOR = 1.96, [95%CI: 1.33, 2.88], Higher education: aOR = 2.80, [95% CI: 1.51, 5.19]) (Lamichhane et al., 2024).

The findings of our study demonstrate that economic and educational inequalities are similarly oriented and are explained by the approachability/acceptability (information via CHWs, female provider preference, hesitancy to seek care unaccompanied) and availability/affordability (proximity, transport, ability to pay) factors. Major accessibility barriers were identified from the χ^2^ test of independence on the observed data, as well as permutation importance and SHAP after predictive analysis. These factors were a lack of access to service information, the disempowered status of women, hesitancy in unaccompanied care seeking, lack of proximity or transport facilities, financial constraints, and perceived quality of care. In other words, findings, like women reporting no accessibility barriers considered in the framework, reveal higher screening rates. This aligns with previous evidence synthesis and observational studies that identify similar supply- and demand-side barriers across LMICs, particularly in India (Almohammed, 2024; Grosse Frie et al., 2013; Mahalakshmi and Suresh, 2020).

CI estimates reveal substantial pro-rich, pro-educated screening uptake, indicating that economic and educational deprivation shape the uptake. Conversely, relatively higher screening uptake among the socially disadvantaged subgroup might reflect targeted efforts aimed at caste-based disparities. Availability and accommodation, acceptability, approachability, quality of care, and spatial access-related barriers steepen the economic and educational gradients. Our finding is also supported by the literature review by Almohammed (2024), which revealed a recurring pattern of obstacles related to awareness, cultural barriers, unavailability of female staff, transport, and cost concerns (Almohammed, 2024). Targeted interventions to enhance affordability, acceptability, availability, and accommodations might be favoring screening uptake among scheduled castes and tribes; however, the decomposition reveals a negligible contribution of the factors considered in the theoretical framework. It demands further detailed investigation related to caste-based inequality predictors in the future.

The results of SHAP analysis reveal that spatial and structural determinants—predominantly state of residence (0.110), rural–urban location (0.100), and autonomy in healthcare seeking (0.064)—are the most influential predictors of breast cancer screening uptake. This emphasizes the role of subnational disparities and women’s autonomy in seeking preventive health services. This is similar to the findings in Patil et al. (2023) and Negi and Nambiar (2021), who indicated that subnational disparities, infrastructural limitations, constrained decision making power, and financial access critically shape preventive health behaviors in India (Negi and Nambiar, 2021; Patil et al., 2023). Our model also reveals that interaction with community health workers (CHWs) (SHAP = 0.053) and less autonomy in seeking unaccompanied care (SHAP = 0.039) exert comparable effects, emphasizing the role of informational and psychosocial empowerment of women in bridging gaps in screening uptake. Our finding is consistent with Rahman et al. (2025), who substantiated education (0.056) and socioeconomic status (0.034) as fundamental drivers (Rahman et al., 2025).

In a comparative analysis of traditional and ML-based inequality estimates, the ML-based concentration indices revealed a better comprehension of inequality patterns than linear techniques did. The Ordinary Least Squares-based concentration indices showed strong pro-rich (0.168) and pro-educated (0.261) inequality in breast cancer screening uptake, whereas the Decision Tree–based concentration indices were comparatively lower (0.108 and 0.182, respectively). This observed difference suggests that a significant portion of the socioeconomic disparities is mediated through the adjustable interdependencies among systemic factors such as autonomy, affordability, and proximity. These findings are in accordance with those of O'Donnell et al. (2008) and O'Donnell et al. (2016), who suggest caution in using linear CIs, which, by ignoring non-linear and interaction effects, may overestimate inequality (O'Donnell et al., 2008; O'Donnell et al., 2016). Similar considerations were documented in ML-based inequality research in maternal health in sub-Saharan Africa, underscoring the higher generalizability of ML-based decomposition in equity analysis (Zegeye et al., 2025).

This understanding is further enhanced by the DT-based application of SHAP decomposition to CIs as found in Candio (2024) and Zhu et al. (2024). Rural location (−0.009), limited autonomy (−0.010), and distance to facilities (−0.005) depict pro-poor CI features as well as negative contributions, indicating that higher targeting of poor, disempowered, rural women living in hard-to-reach areas is likely to reduce inequality. On the other hand, education and economic status were the primary positive contributors to the disparity in economic and educational rankings, and CI features are pro-rich and pro-educated. Additionally, disparity can be reduced by enablers such as CHW interaction (−0.001, −0.0003) and empowerment (−0.010 and −0.012) when economic status or education was used as the ranking criterion. However, access to water (0.003, 0.002) and sanitation (0.002, 0.001). and CHW engagement (0.003, 0.005) depicting pro-richness, indicating poor families are still deprived of these services. This indicates that community outreach and empowerment alleviate the structural burden of social hierarchy if properly targeted at poorer and less educated women, supporting the findings of similar studies (Mainaz et al., 2024; Palaniraja et al., 2025). In a nutshell, feature-specific CI contributions show that the burden of limited proximity, transport, hesitancy barriers, affordability barriers, and reduced autonomy falls disproportionately on disadvantaged women. These patterns were consistent across economic and education-based subgroups. However, when inequality was ranked by ethnicity, the same factors indicated the need for further in-depth research. This reflects a mixed empirical pattern, and this study recommends further research to identify the mediators and moderators under access barriers that align strongly with caste divisions. Overall, these observations confirm the robustness of directionality even when subgroup CI magnitudes varied.

Our findings contrast with some previous research that predominantly considered wealth or education as the primary contributors to inequality (Rahman et al., 2025; Sen et al., 2022). Although these are powerful predictors, the current analysis shows that inequalities are multidimensional and mediated by various structural and behavioral aspects. The lower CI magnitudes in ML-based models suggest that, rather than being solely due to economic disparities, some of the “wealth effect” observed in non-linear analysis really represents fundamental barriers to empowerment and accessibility. This question challenges the oversimplified narrative of socioeconomic theories. It emphasizes the need to incorporate psychosocial and structural factors into frameworks that account for basic social, economic, and educational equity gradients in a nonlinear manner. Therefore, as evident from the study of Chandar et al. (2025), the present study shows enhanced prediction of uptake/non-uptake of breast cancer screening programs in rural India, identifying those less likely to attend the programs, which can enhance CHW’s role in management to alter the screening preference of at-risk middle-aged women through positive deviance.

### Limitations

The study has several limitations. The use of cross-sectional data (NFHS-5) limits the ability to explore temporal variation to draw causal inferences about the trend of screening behavior. Secondly, self-reported data on ever undergoing breast cancer screening may be subject to recall bias with the possibility of social desirability bias, potentially affecting the actual coverage estimate. Although AUROC values for XGBoost and RF were close to 1.00 after SMOTE oversampling, these may reflect overfitting. Cross-validated DT performance was more stable and thus more reliable for inequality estimation. Thus, classifier performance across 10 splits identified DT as the most stable model for screening prediction, thereby mitigating the risk of overfitting risk-affected inference. Fourth, CI decomposition relies on country-level averages of factors under the Levesque framework. It may have a confounding effect due to unobserved factors such as regional or locational variation in community-specific traits, which need to be explored using an inductive approach and complement the future aim of multilevel decomposition. In the future, it needs to be adopted to understand the caste-based disparity in screening uptake. Qualitative findings will reveal the contextual nuance of cultural facets influencing screening behavior. Nevertheless, advanced analytics applied provide a strong scientific foundation for policymakers to identify socioeconomic inequities and the structural and behavioral mediators shaping disparities in preventive health uptake that are driving downward trends in coverage in India.

### Future research directions

We aim to conduct a longitudinal study using mixed-method approaches to explore the temporal pattern and causal pathways based on contextually relevant accessibility barriers affecting screening behavior. Integration of facility and community-level data would map supply- and demand-side contributors to spatial inequity and reveal district/block/local self-governance-level disparities. Joining survey data with routine health information management data can strengthen model generalizability and reduce reporting bias. In this fashion, we will be able to investigate how community-level public health interventions integrate digital outreach involving women’s health collectives such as self-help groups and CHWs to mediate screening inequalities. Furthermore, machine learning pipelines would include fairness-aware explainable AI methods to enhance transparency and algorithmic equity. The objective is to build a consortium of public health researchers, data scientists, and the local health systems to translate predictive insights into actionable screening equity strategies. Such evidence-driven interventions optimize community health workers’ responsibilities by feeding into state-level policy design. The new strategy will emphasize the equitable participation of poor, less educated, and socially marginalized women in cancer screening programs.

## Conclusion

This study demonstrated that machine learning–driven prediction can efficiently identify predictors of breast cancer screening uptake and inequalities in uptake in India. Economic, educational, and social gradients shape screening participation, while accessibility, autonomy, CHW contact and service discussions, and hesitancy factors act as crucial mediators of inequity. The ML-based CI decomposition analysis highlights that financial and spatial barriers disproportionately affect poorer and less educated women, while targeting through informational and psychosocial enablers, e.g., CHW-led discussions and empowering them to make decisions, can alleviate disparities. The methodological integration of explainable AI and the concentration index, along with learner-based CI decomposition, brought methodological advancements. It generated more accurate, precise, and actionable insights into complex inequity structures that impede preventive care uptake. Findings underline the demanding need for multisectoral involvement to mitigate affordability, proximity, and empowerment gaps at the community level. Rebuilding awareness through data-driven targeting can eradicate inequities and advance equitable cancer prevention and control in resource-scarce settings.

## Data Availability

The data that support the findings of this study are available from: [https://dhsprogram.com/data/available-datasets.cfm].
