# Supplementary material for: Predicting and identifying correlates of inequalities in breast cancer screening uptake using national level data from India
Source: Front Artif Intell. 2026 Jan 20;8:1729796. doi: 10.3389/frai.2025.1729796 (PMC12820423; doi:10.3389/frai.2025.1729796)
Supplement: Supplementary file 1 [file Data_Sheet_1.docx]

Supplementary Table I. CLAIM Checklist and Study Compliance

| **CLAIM Item** | **Description** | **Where Addressed in Manuscript** |
| --- | --- | --- |
| Data source | Describe dataset origin, population, inclusion/exclusion | Methods → NFHS description |
| Preprocessing | Handling of missing data, balancing, transformations | Methods → Data preprocessing (SMOTE, weighting) |
| Model development | Algorithms used, hyperparameters, train-test split | Methods → Predictive analysis |
| Model evaluation | Metrics (AUROC, accuracy, F1), cross-validation | Methods → Evaluation metrics; Results → Table 3 & 4 |
| Model explainability | SHAP, permutation importance | Methods → Feature importance; Results → Figure 3, Table 6 |
| Fairness considerations | Inequality analysis with CI/ML decomposition | Methods → CI & decomposition; Results → Table 7 & 8 |
| Limitations | ML interpretation constraints and overfitting | Discussion → Limitations |

**Concentration Index:**

The Concentration Index (CI) was employed to measure socioeconomic inequalities in access to breast cancer screening at all India level. The CI quantifies the extent to which access to screening is distributed unequally among individuals with different socioeconomic and demographic characteristics, such as economic status, educational attainment, caste, and age. The general formula is:

$$\mathrm{CI} = \frac{2}{\mu}\cdot\text{Cov}\left( y_{i}, R_{i} \right)$$

Where:

- $y_{i}$= the outcome variable (breast cancer screening) for individual *i*
- $\mu$= the mean of $y$ across the population
- $R_{i}$ = the fractional rank of individual *i* in the socioeconomic distribution (e.g., ranked by wealth or income, from poorest to richest, by age from younger to older, by education level and by ethnicity)
- $\text{Cov}\left( y_{i},R_{i} \right)$= the covariance between the health variable *y* and the fractional rank *R*

The CI is defined as twice the area between the concentration curve and the line of equality (the 45-degree line). The concentration curve plots the cumulative proportion of women who reported undergoing breast cancer screening (on the y-axis) against the cumulative proportion of the population ranked by a given socioeconomic variable — such as wealth (from poorest to richest), education (from no education to higher education), caste (from lower to upper groups), and age (from youngest to oldest) — on the x-axis. A CI value of 0 indicates perfect equality; negative values indicate that screening access is concentrated among disadvantaged groups, while positive values suggest concentration among more advantaged populations(Adam Wagstaff, 2008).
